# 

**Published:** 1874-01

**Authors:** 


### CONTENTS.

A

| | PAGE |
|---|---|
| Accidents, Uniformity in | 251 |
| America Leads | 399 |
| American Girl Abroad | 205 |
| Anæsthetics | 183, 276 |
| Animals and Plants | 360 |
| Animal Teaching | 157 |

B

| | |
|---|---|
| Babies, Cruelty to | 434 |
| Backbone | 71 |
| Bad Temper and Insanity | 118 |
| Beards | 74 |
| Bed, a Wholesome | 461 |
| Beef Tea | 229 |
| Bees | 82 |
| Beverages | 299 |
| Biliousness | 377 |
| Blood, The | 376 |
| Boils, Remedy for | 259 |
| Boots and Shoes, Construction of | 361 |
| Brain, The | 257 |
| Bronze Printing | 280 |
| Bunion | 35 |
| Burying Alive | 430 |

C

| | |
|---|---|
| Camphor Medication | 190 |
| Cancer | 233 |
| Carbolic Acid | 465 |
| Carbuncle, Cure of | 259 |
| Cellars | 457 |
| Cellars, Freezing | 341 |
| Chilblains | 34, 190 |
| Children, How to Save | 477 |
| Cholera, Causes of | 150, 240 |
| Choose—Health or Sickness | 304 |
| Cleanliness | 50 |
| Clergymen Warned | 62 |
| Coal | 302 |
| Colds, Borax for | 84 |
| " Cure of | 116, 274, 375 |
| Cod-liver Bread | 339 |
| Closets | 235 |
| Color | 280 |
| Combustible, the New | 104 |
| Constipation | 465 |
| Constitution, Hardening of | 119 |

| | PAGE |
|---|---|
| Consumption, earliest Sign of .. .. .. | 69 |
| Corrosive Sublimate—Antidote .. .. .. | 115 |
| " " detects Counterfeits .. . | 115 |
| Corns .. .. .. .. | 37, 142 |
| Cough Medicines .. .. .. .. | 375 |
| Crime .. .. .. .. .. | 263 |
| Crushed White Wheat .. .. .. | 452 |
| **D** | |
| Death at Watering-places .. .. .. | 266 |
| " Signs of .. .. .. .. | 375 |
| Desserts .. .. .. .. .. | 438 |
| Dieting for Health .. .. .. .. | 117 |
| Diptheria .. .. .. .. | 464 |
| Diptheria and Scarlet Fever .. .. .. | 294 |
| Disastrous Times .. .. .. .. | 1 |
| Diseases, Spread of .. .. .. .. | 454 |
| Disinfectants .. .. .. | 292, 337 |
| Dreams .. .. .. .. .. | 85 |
| Drinks, Healthful .. .. .. .. | 232 |
| Dyspepsia .. .. | 232, 309, 368, 378, 425 |
| **E** | |
| Early Rising .. .. .. .. | 268 |
| Ears, Boxing the .. .. .. .. | 379 |
| Eating too Little .. .. .. .. | 465 |
| Emanations .. .. .. .. | 261 |
| Eye, Diseases of .. .. .. | 52, 273 |
| " How Swept and Washed .. .. .. | 355 |
| Eyes and Cold Water .. .. .. .. | 114 |
| " Different kinds of .. .. .. | 103 |
| **F** | |
| Fall Sickness .. .. .. .. | 378 |
| Fever and Ague Destroyer .. .. .. | 50 |
| Felons .. .. .. .. .. | 228 |
| Fireside, Cosy .. .. .. .. | 463 |
| Feet, Cold .. .. .. | 19, 64 |
| " Distortions of . . .. .. | 448 |
| " How to Clothe .. .. .. .. | 466 |
| Food .. .. .. | 14, 36, 302, 499, 463 |
| " Adulteration .. .. .. .. | 401 |
| " Cheap .. . .. .. | 35 |
| " Nourishing .. .. .. .. | 309 |
| Frost Bite .. .. .. .. | 33 |
| Fuel, A Remarkable .. .. .. .. | 452 |
| " Economy in .. .. .. .. | 38 |
| Furnace, Gothic .. .. .. .. | 477 |
| Fussing .. .. .. .. .. | 435 |
| **G** | |
| Gas, How made .. .. .. .. | 242 |
| Gipsies. Origin of .. .. .. .. | 98 |

PAGE.

Glencove .. .. .. .. 404
Going Abroad .. .. .. .. 221
Grand Rendezvous, The .. .. .. 23
Grapes .. .. .. .. 340, 377
Green Scum .. .. .. .. 296

H

Habit .. .. .. .. .. 72

Happiness .. .. .. .. 122

Hecker's Farina .. .. .. .. 452

Health Resorts, .. .. 76, 130, 175, 195, 255, 323

" of Countries .. .. .. .. 372

Heat in Disease .. .. .. .. 471

Home Adornments .. .. .. .. 391

" Influences .. .. .. .. 173

Hotels .. .. .. .. .. 412

House-building .. .. .. 109, 185

" -heating .. .. .. 300, 402, 474

Houses, Healthy .. .. .. 105, 429

" New .. .. .. .. 31

Human Locomotion .. .. .. .. 454

Humboldt .. .. .. .. 203

Hydrophobia .. .. .. 94, 239, 308

I

Ice Houses, Cheap .. .. .. .. 338
Imagination Power .. .. .. .. 329
Inebriate Asylums .. .. .. 298, 330
In Case of Fire .. .. .. .. 437
Infant Prodigies .. .. .. .. 207
Insanity and Suicide .. .. .. .. 29
Insects .. .. .. .. .. 376

J

Japanese Paper Factory .. .. .. 207
" Women .. .. .. .. 162

K

Kings Nursing Fathers .. .. . 32
Kitchen, The . .. .. .. 86

L

Lamp Dangers .. .. .. .. 267
Land-mark, Metropolitan .. .. .. 419
Latitude at Sea .. .. .. .. 281
Laughing .. .. .. .. 234
Lead Pipes and Water .. .. .. 307
Lessening Production .. .. .. 441
Life, Duration of .. .. .. .. 73
" Insurance .. .. .. .. 441
" Localities of .. .. .. .. 428
Linoleum .. .. .. .. 446
Longevity, Law of .. .. .. .. 356

M

| | PAGE. |
|---|---|
| Man and the Locomotive | .. 460 |
| Matches | .. 331 |
| McComber's Lasts | 392, 479 |
| Meats, Baking | .. 87 |
| Medication by Absorption | .. 484 |
| Memoranda, Scientific | .. 208 |
| Memory of Faces | .. 172 |
| Mind and Health | .. 111 |
| " and Milk | .. 27 |
| Miscellaneous | 473 to 484 |
| Modern Match Making | .. 152 |
| Moon Coal | .. 335 |
| Mortality of Cities | 119, 121 |
| Mountain Air Everywhere | .. 139 |

N

| | |
|---|---|
| Nature in the Parlor | .. 170 |
| New Homes | 124, 178 |
| Ninety Years Old | .. 365 |
| Not Lost | .. 311 |
| Nurses | .. 236 |

O

| | |
|---|---|
| Oat Meal | .. 149 |
| Onions | .. 39 |
| Open your Windows | .. 472 |
| Oyster Cultivation | .. 454 |
| Ozone | .. 139 |

P

| | |
|---|---|
| Patents | .. 456 |
| Pepsin Preparations | .. 189 |
| Peruvian Bark | .. 472 |
| Piles Cured | .. 36 |
| Plants, Sleep of | .. 87 |
| " Ways of | .. 354 |
| Plant a Tree | .. 338 |
| Poisons | 230, 295 |
| Poisonous Wall-paper | 53, 144 |
| Power, Source of | .. 188 |
| Preacher, The Unwise | .. 24 |
| Preservative | .. 53 |

R

| | |
|---|---|
| Rats, How to Banish | .. 472 |
| Reading Aloud | .. 260 |
| " Desultory | .. 154 |
| Religion of Health | .. 413 |

S

| | |
|---|---|
| Salt in Sickness | .. 142 |
| Savings Banks | .. 451 |
| Scarlet Fever | .. 294 |

| | PAGE. |
|---|---|
| Sea-sickness | 85 |
| Sedentary Habits | 102 |
| Scandinavia | 205 |
| Sex, Equality of | 357 |
| " in Education | 370 |
| Sight | 93 |
| Singers, Public | 241 |
| Sleep and Death | 75 |
| Sleepless Nights | 226 |
| Sleeping Rooms | 459 |
| Spectacles | 227, 433 |
| Sprains | 35 |
| Starving, Sensations of | 243 |
| Steinway, House of | 447, 470 |
| Stimulants | 426 |
| Strength, Economy of | 432 |
| Sugar | 297 |
| Sweetmeats | 144 |
| Syrups | 277 |
| **T** | |
| Tasteless Medicines | 418 |
| Teeth, Decay of | 305 |
| " How Preserved | 389 |
| Throat Ail | 54 |
| Thumbs | 96 |
| Tidy Surroundings | 366 |
| Tongue, Uses of | 49 |
| Trees in Winter | 128 |
| Trichinosis | 434 |
| Tobacco | 463 |
| " Smoking | 455 |
| **U** | |
| Urine, Bloody | 189 |
| **W** | |
| Walking | 377 |
| Water Closets | 460 |
| Water Dressings | 434 |
| " Drinking | 147 |
| " Snow | 159 |
| Weather Changes | 30 |
| " Signs | 155 |
| Wells | 374 |
| Wheat-meal | 84 |
| Winter Wear | 431 |
| " Wisdom | 17 |
| Wood | 39 |
| Woman as a Physician | 416 |
| Work and Worry | 143 |
| Poetry and Miscellany. | |

### BOOK NOTICES.

MAKE A BOOK OF IT.—*The Christian Observer*, of Louisville, Kentucky, has had a contributor for a year past who writes from "Farndale, Kentucky," on the subject of Language. These articles are too valuable to be allowed to pass away with a weekly newspaper; they would make a book which would be read with exceeding interest, and studied by professional men and scholars all over the country. The writer, "J. S. B.," is master of his subject, and wields the pen of a ready writer.

THE MAGIC INKSTAND.—This is the very appropriate title of a new invention just introduced into this country, and for sale by Messrs. Root, Anthony & Co., of Liberty Street. It is patented in France and Great Britain by the eminent book publishers, Messrs. Hachette & Co. (Paris), and Sampson Low & Co. (London). Fifty thousand of these useful articles have been sold in Europe in less than six months. It is a perfect marvel of economy, utility, durability and simplicity. The Magic Inkstand produces ink of the best quality in every desirable color—ink, moreover, which is not affected by acids, climate or temperature, which does not oxydize the pen (a valuable feature), and which leaves no sediment. It is made in a few minutes, and is always renewable simply by the addition of pure water. It is well adapted for use in the counting house, office, school room or parlor. For sale by all stationers and booksellers; price, two dollars.

GARDENING.—James Vick, of Rochester, the Napoleon of seed and nurserymen, has issued his FLORAL GUIDE for 1874. A most beautiful annual, 25 cents, containing a very large amount of useful information pertaining to floriculture and gardening generally, with very many beautiful illustrations.

*Expression: Its Anatomy and Philosophy.* By Sir CHARLES BELL, K. H. With notes, and upward of seventy Illustrations. Tinted paper, fancy muslin, beveled boards. 12mo, pp. 200. Very handsome. Price, $1.50. New York: S. R. WELLS, publisher.

This volume is the expression of a mind of high culture and very comprehensive observation. It must be universally acknowledged as a work of standing authority in the departments of Art and Science to which it belongs. In this new addition much new matter has been added by the editor, and additional illustrations and notes have been introduced to render the work complete and attractive.

*The Attraction of the Cross.* By Rev. GARDINER SPRING, D. D., New York City. The best of Dr. Spring's works, on the noblest of themes, illustrating the leading truths, obligations and hopes of Christianity. It unfolds the doctrines of the Cross as the only propitiation for sin, defends it against assaults and objections, and shows the fullness of its power to save, and the assurances of its final triumph. 413 pages, large 12mo, $1. Postage 20 cents. American Tract Society, 150 Nassau street, New York.

"*The Fruit Recorder and Cottage Gardener*," published monthly at one dollar a year by A. M. PURDY, Editor, Palmyra, New York, contains a large amount of practical information in reference to the cultivation of fruits and flowers. Intelligent subscribers will be richly repaid for their outlay.

AUTHORS, NEWSPAPER REPORTERS and CLERGYMEN will find at the Morgan Envelope Co., at Springfield, Mass., what they will consider a treasure, and so will that immense multitude composed of those who do not want to write long letters and those who do not like to be compelled to read long ones.

They manufacture a paper in strips of four inches by eight, five by ten and six by twelve. It is every way preferable to the old fashioned folded note paper, less cumbersome, more handy and more economical. In most letters and short notes one half the writing paper is wasted. Merchants seldom write on more than one page of the four. To type-setters it is an incalculable benefit as compared to manuscript which has to be set up, it covers but four inches broad of the case, instead of double. For five dollars the firm will send specimens of the different sizes, sermon papers, and one of the most convenient and perfect glass inkstands, with a glass gum-holder and brush; the very thing for editors and all who have a predilection for putting away useful scraps.

"*Young Folks' Journal,*" *for December.* $1 a year. We bespeak a wide circulation for *Young Folks' Journal*, and success for the family of girls who edit and publish it. Send a stamp to Lukens Sisters, Brinton, Pa., for a specimen copy, and read their Club and Premium Lists.

"*How? How can it be done?*" *The Interior* published weekly at Chicago and St. Louis, is one of the largest and best conducted religious newspapers in the whole Presbyterian Church, $2.50 a year, and yet it offers a five dollar book, with *The Interior*, for Five Dollars—the book itself being one of the very best issued by the religious press within the last ten years, to wit, "Smith's Bible Dictionary," 8vo. *The Interior* is earnestly commended to the patronage of the great Northwest, especially of Presbyterian families.

*Something New!!*—A secular newspaper which is never against religion, the clergy, the Bible, or the Sabbath-day; which can always be admitted into families of refinement and cultivation, without any misgivings—its fair columns never defaced by vulgarities, profanities, infidelities and the slang of the street, and yet it gives the latest political, and general, and financial news, besides a very large amount of family reading, interesting and instructive to old and young. Reference is here made to

"*The New York Weekly Witness,*" a large sheet, well filled, at the very low price of one dollar a year. It is a paper which should be patronized by the subscriptions and advertisements of merchants, bankers and business men in general as a matter of princple, for thereby they aid in sustaining a paper which disseminates a vast amount of valuable, instructive, moral reading among the masses, at a cost of less than two cents a week! Its circulation is now rapidly reaching twenty thousand copies weekly, to actual subscribers, and is supplanting the trashy newspapers in very many places. As to the safe and unexceptionable reading matter of *The New York Weekly Witness*, there is great satisfaction in being able to say of three other dearer publications, that in our long experience as an editor—over twenty years exchanges—there never has been noticed in any one of them, not in any page or paragraph, or line, one single expression or sentiment, remotely antagonistic to the Christian religion. We mean, in alphabetical order—*Godey's Lady's Book*, of Philadelphia, *Home Journal*, of New York, and the *Scientific American*, published by Munn & Co., 37 Park Row, New York City; and let their editors have their deserved honors for the same, manifested by an increasing patronage on the part of all cultivated minds. It is a positive delight for us to be able to write thus from our own knowledge. It ought not to be omitted to state that by the course they have pursued, all these publications have had a steadily increasing prosperity, and have made money largely.

### SOME EXCHANGES OF GENERAL INTEREST.

### MAGAZINES.

*Atlantic Monthly.* $4 a year. Published by HURD & HOUGHTON, 13 Astor Place, Eighth street, New York, and H. O. HOUGHTON & Co., Boston, Mass. The writers for this well known magazine are of the highest talent and ability, causing it to be sought for and patronized by the best educated men in the country. Now in its 33d volume.

*Harper's Magazine*, issued monthly at $4 a year, with a subscription list of one hundred and thirty thousand copies and over, attests the hold it has on the appreciation of the public. It is profusely illustrated, is always got out in time and in handsome style, at great cost. The reading matter is always varied, instructing and elevating. In addition to a large amount of general reading, there are many pages given to items in science, art, politics and general news and history. Now in its 48th volume.

"*Harper's Bazar of Fashion.*" Issued monthly at $4 a year, and *Harper's Illustrated Weekly*, $4 a year. New York.

"*Lippincott's Magazine.*" An illustrated monthly of popular literature and science. Each number contains numerous illustrations. $4 dollars a year; single numbers 35 cents. Published by J. P. LIPPINCOTT & Co., Phila.

"*Scribner's Monthly.*" $4 a year. New York. Has for its contributors some of the very best writers of the times. Liberally illustrated with drawings taken from nature, historical, &c. *Scribner's* is always greatly prized by its readers and every number is ably edited.

"*The Journal of Applied Chemistry*" is published monthly simultaneously in Boston, New York and Philadelphia. 16 pages quarto, $2 a year. A very valuable publication for chemical manufacturers, druggists, apothecaries and physicians. Every number has several pages of well selected general matter always useful and interesting.

"*Boston Journal of Chemistry.*" Monthly. $1.50 a year. Well deserves a place on the table of every householder. In spite of its scientific name, it is specially adapted to all families, as it contains a great deal of reliable information on matters of domestic comfort and economy. Not a single number is issued which does not communicate the knowledge of various practical matters, which if not elsewhere obtained, is worth more than a year's subscription. Let any one try it for three months for fifty cents.

"*Druggists' Circular.*" 36 Beekman street, New York. $1.50 a year. issued monthly. Now in its 18th volume. Is always valuable, not only to druggists and apothecaries, but to physicians, old and young, and of all schools, giving early notice of new medicinal agents, surgical instruments, etc.

"*Herald of Health.*" $1.50 a year. Published by WOOD & HOLBROOK, 13 and 15 Laight street, New York; is devoted to the culture of body and mind. Its motto being, "A higher type of manhood, physical, intellectual and moral." It has done the public good service these many years.

"*Wilson's Herald of Health.*" Published quarterly at Atlanta, Georgia. 43 Whitehall street. JOHN STAINBACK WILSON, M. D., Editor. Devoted to the diffusion of health preserving knowledge among the people. It is well worthy of an extended patronage. The two numbers are full of good things.

"*New York Weekly Witness.*" New York. $1 a year. The safest and best dollar newspaper weekly published in the world, and every man and woman who patronizes it by subscription and advertisements does a public good.

